# Neonatal pain response to automatic lancet versus needle heel‐prick blood sampling: A prospective randomized controlled clinical trial

**DOI:** 10.1111/ped.14142

**Published:** 2020-04-03

**Authors:** Tatenobu Goto, Takeshi Inoue, Chinami Kamiya, Hiroyuki Kawabe, Machiko Higuchi, Megumi Suyama, Tomoki Goto, Wakato Koide, Kanemasa Maki, Katsumi Ushijima, Kyoko Ban, Yasumasa Yamada

**Affiliations:** ^1^ Division of Pediatrics Yokkaichi Municipal Hospital Yokkaichi Mie Japan; ^2^ Department of Perinatal and Neonatal Medicine Aichi Medical University Sciences Nagakute Aichi Japan

**Keywords:** automatic lancet, blood sampling, heel prick, neonate, pain

## Abstract

**Background:**

Automatic lancets have been reported to be superior to manual lancets in terms of pain and treatment time. However, no studies have yet been published comparing automatic lancet and needle puncture heel‐prick blood sampling. The objective of this study was to compare the pain response and efficiency between the automatic lancet and needle at the time of heel blood sampling. The design was a randomized controlled trial. The inclusion criteria for the participants were a birthweight of ≧1,500 g and a gestational age of ≧30 weeks.

**Methods:**

The study examined a total of 105 neonates who were randomized into an automatic lancet group (*n* = 53) and a needle group (*n* = 52). The parameters measured included blood collection time, number of calf squeezes, duration of audible crying, and the Neonatal Infant Pain Scale (NIPS) score. The main outcome measure was audible crying duration.

**Results:**

The duration of audible crying was significantly shorter in the automatic lancet group when compared to the needle group (median 3 s, interquartile range (IQR) 0–33 s vs median 39 s, IQR 5–91.5 s, *P *= 0.0023). The NIPS score at the time of puncture was significantly lower in the automatic lancet group than in the needle group (median 1, IQR 0–5 vs median 5, IQR 3–6, *P *= 0.0060). There was no significant difference in the blood collection time and the number of calf squeezes between the two groups. The automatic lancet was found to be less painful than the needle puncture in neonatal heel‐prick blood sampling with no significant difference in blood sampling time.

**Conclusion:**

The automatic lancet was found to be less painful than the needle puncture in neonatal heel‐prick blood sampling with no significant difference in blood sampling time.

Blood sampling by heel prick collection is the most common painful procedure performed on neonates. Heel blood samples are commonly used for biochemical evaluation, blood gas analysis, and neonatal screening tests. For this reason, infants admitted to the neonatal intensive care unit (NICU) may undergo multiple heel pricks during this period. Neonates admitted to the NICU may undergo up to 10 painful treatments per day of hospitalization, and pain relief is often not considered.^1^ Repeated pain exposure during periods of rapid neurodevelopment has been shown to alter brain microstructure^2^, ^3^, ^4^ and risks long‐term adverse effects on emotional, behavioral, and cognitive development.^5^, ^6^

Neonatal pain is still underestimated in NICUs, largely due to the fact that neonates cannot complain of pain themselves and are often believed less likely to experience pain by medical staff. If neonatal pain is managed properly, it can minimize many short‐ and long‐term negative outcomes, including emotional and behavioral disturbances as well as learning impairment.

The heel‐prick procedure is a relatively simple procedure, but infant pain, bruising and, rarely, severe complications such as osteomyelitis, are problems. Heel blood collection via needle puncture is carried out in many NICUs, especially in Japan,^7^ and is both cost effective and easy to perform. Some studies have suggested that automatic lancets reduce blood collection time, need for squeezing, heel wounds, bruising, pain, and the need for repeated heel puncture.^8^, ^9^, ^10^, ^11^, ^12^, ^13^ It has previously been reported that manual lancets were superior to needle puncture in reducing pain.^14^ Several papers have compared automatic lancets with manual lancets, but there is currently no published data comparing automatic lancets with needle puncture in terms of pain response, time taken to complete the blood collection, and the presence of bruising.

The main purpose of this study is to compare the pain response and efficiency between the automatic lancet and needle at the time of heel blood sampling.

In Japan, a mass screening system has been established in order to detect inherited metabolic diseases. Approximately 100% of neonates in Japan undergo this mass screening,^15^ where blood obtained by heel puncture is used to fill four dried blood spots 10 mm in diameter on a filter paper between 4 and 7 days after birth. We planned to use this mass screening test as an opportunity to compare the automatic lancet and needle puncture heel blood sampling techniques in a randomized controlled trial.

## Methods

This study was designed as a prospective, randomized controlled clinical trial. Hemodynamically stable infants admitted to the NICU and undergoing a heel prick for the routine newborn screening test at between 4 and 7 days old were eligible for inclusion in the study. Inclusion criteria were a birthweight of 1,500 g or more and a gestational age of 30 weeks or more. Those on ventilator support, those with neurological abnormalities or congenital anomalies, and those with exposure to analgesic medications, were excluded from the trial.

Sample size was calculated based on previous studies of pain comparison in heel blood sampling and required 50 cases in each group for the study to have a power of 80% with an alpha error of 0.05.

Once written consent was obtained from the parents, eligible neonates were randomized into two groups using a computer‐generated randomized sequence. Infants were assigned to either the automatic lancet group or the needle group just before heel sampling. The concealment of the assignment was achieved by sequentially removing opaque seals covering the computer‐generated randomized sequential numbers. Ethical approval was granted by the Ethics Review Committee of Yokkaichi Municipal Hospital (No. 2017‐22).

The following instruments were used: A 23‐gauge needle (Terumo Needle 23G × 1” NN‐2325R, Terumo Corporation, Tokyo, Japan) was compared with an automated heel‐lancing incision device with a 1 mm deep and 2.5 mm long blade (BD Microtainer Quickheel Lancet, Beckton Dickinson Company, Franklin Lakes, NJ, USA). We used two types of devices, a 23‐gauge needle and an automatic lancet, at the time of this study and selected one of them for heel blood sampling according, as decided by blood samplers.

Heart rate (HR) was measured using the electrocardiogram trace obtained from the heart rate monitor (IntelliVue MP50, Phillips Medical Systems, Boeblingen, Germany). Percutaneous arterial oxygen saturation (SpO_2_) was continuously recorded via a pulse oximeter probe connected to the lower extremity of the neonate and a monitor (IntelliVue MP50, Phillips Medical Systems, Boeblingen, Germany). Heart rate and oxygen saturation measurements were recorded at two points during the procedure: just before and 1 min after heel prick.

All infants were fed between 1 and 1.5 h before the heel puncture. Their diapers were changed, and each was placed in the supine position 30 min before the heel puncture procedure. The examiner was one of 11 doctors, each with between 2 and 28 years of clinical experience and they participated after training with both devices for at least two weeks.

The heel blood sampling was conducted according to the following procedure. The heel was cleaned with an alcohol swab and then punctured with either a 23 G needle or the automatic lancet device. The heel was then repeatedly squeezed until all four spots of the filter paper were stained with blood. At the end of blood collection, the puncture site was again wiped with an alcohol swab and covered with an adhesive bandage.

This study was conducted with environmental strategy including clean diaper, low noise and lightning to prevent and reduce neonatal pain during blood sampling.

The examiner reported the number of squeezes required to fill the four spots of the filter paper after each procedure. None of the samples required re‐testing. The time from the start to the end of blood collection was measured using a stopwatch. Videos of each infant were recorded (GR Digital IV, Ricoh Imaging Co., Ltd, Tokyo, Japan) from before the heel puncture until the end of blood collection, and the Neonatal Infant Pain Scale (NIPS)^16^ score was evaluated later using this video. Four well‐trained, experienced nurses, who were not informed about the group the infant was allocated to and the research object, carried out the video assessment. The audible crying duration was also timed from the recorded video using a stopwatch. Video recordings of the infants' heels were made before heel puncture and at 24 h post blood sampling for the purpose of evaluating bruising, inflammation, and scar formation. The video assessment was conducted by well‐trained neonatologist who was blinded to the allocated groups. Bruising was defined as purpura along the squeezed mark. Inflammation was defined as redness and swelling along the wound. Scar was defined as a liner scar at the time of blood collection.

The primary outcome measure was the audible crying duration. The secondary outcomes were the NIPS score, HR, SpO_2_, and the number of squeezes.

The distribution of the data was tested using the Shapiro–Wilk test. If the data were normally distributed, the *t*‐test was used. If the data were not normally distributed, the Mann–Whitney *U*‐test was used. Data are reported as median values (interquartile range, IQR). Differences were evaluated by the chi‐square test for categorical data. Probability values of less than 0.05 were considered significant. All data analyses were performed with EZR (Saitama Medical Center, Jichi Medical University, Saitama, Japan), which is a graphical user interface for R (The R Foundation for Statistical Computing, Vienna, Austria).^17^

## Results

**Figure 1:**
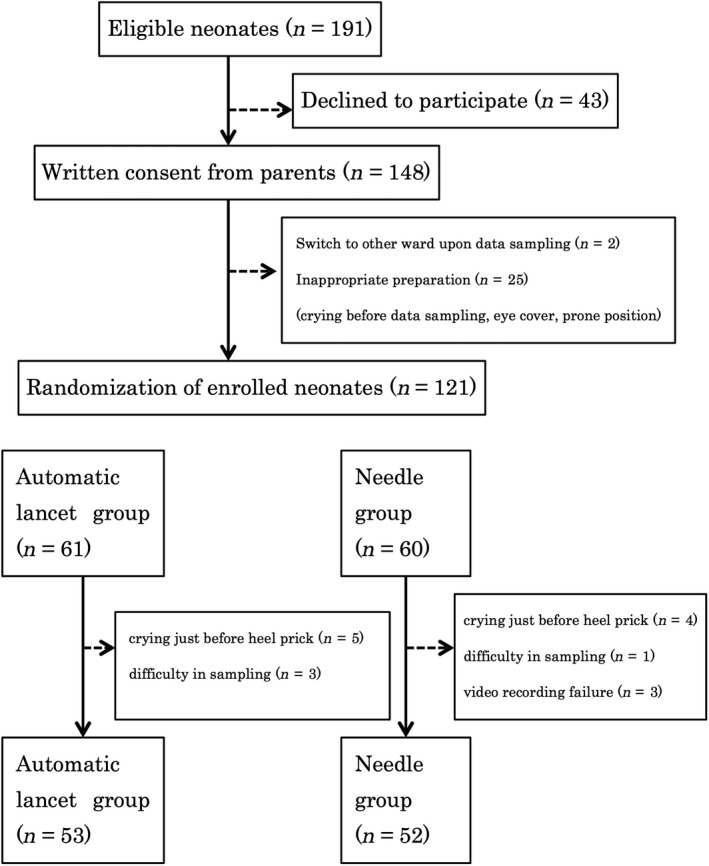
Trial profile.

This randomized controlled trial was carried out in our NICU from November 2017 to September 2018. The study examined a total of 105 infants, assigned to the automatic lancet group (*n* = 53) or the needle group (*n* = 52). The trial flow is shown in Figure 1.

**Figure 2:**
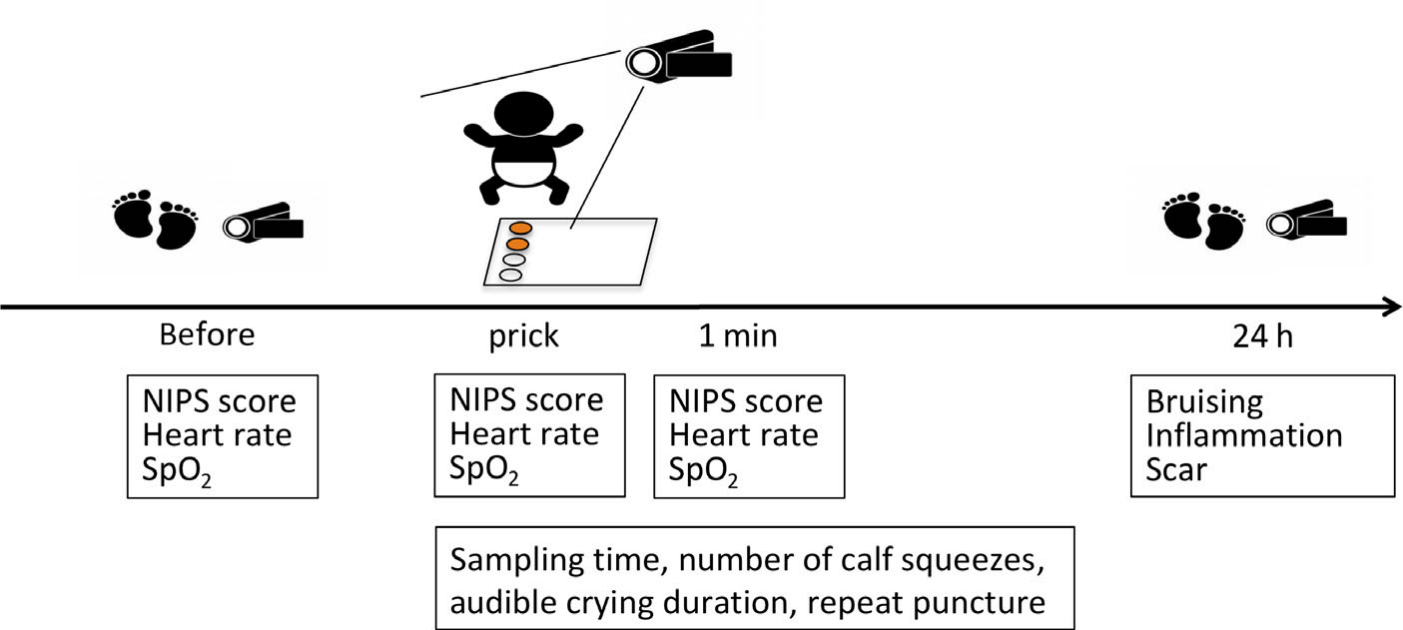
Timeline for measuring the outcome.

Demographic characteristics are shown in Table 1. No statistically significant differences in gestational age at birth, birthweight, Apgar score at 1 and 5 min, sex, and postnatal age at puncture were found between the two groups. Figure 2 shows the timeline for measuring the outcome of this study.

**Table 1:** Demographic characteristics of study subjects

	Automatic lancet (*n* = 53)	Needle (*n* = 52)	*P* value
Gestational age (weeks)	37.4 (36.1–39.4)	37.6 (36.0–39.8)	0.72
Birthweight (kg)	2.48 (2.13–2.98)	2.53 (2.14–3.03)	0.89
Apgar score			
1 min	8 (8–8)	8 (8–8)	0.84
5 min	9 (8.5–9)	9 (8–9)	0.12
Sex (male/female)	25/28	24/28	0.92
Postnatal age (days)	5 (5–6)	5 (5–6)	0.46

Data are shown as median (interquartile range) except for sex.

There was no significant difference in blood collection time – that is, time required to perform the procedure (median 50.0 s, IQR 34–72 s vs median 57.5 s, IQR 37–94 s, *P *= 0.0702) or the number of calf squeezes (median 9, IQR 7–12 vs median 10, IQR 8–14.25, *P *= 0.14) between the two groups (Table 2). The number of cases requiring repeat punctures did not differ significantly between the two groups (automatic lancet group *n* = 3, 5.7% vs needle group *n* = 8, 14.8%, *P *= 0.12). No statistically significant difference in the clinical experience of the examiner was found between the two groups (median 7, IQR 3–16 vs median 7, IQR 3–14, *P *= 0.26).

**Table 2:** Automatic lancet group versus needle group

	Automatic lancet (*n* = 53)	Needle (*n* = 52)	*P* value
Blood collection time (s)	50 (34–72)	57.5 (37–94)	0.070
Number of calf squeezes	9 (7–12)	10 (8–14.25)	0.14
Re‐puncture	*n* = 3 (5.7%)	*n *= 8 (15%)	0.12
Clinical experience (years)	7 (3–16)	7 (3–14)	0.26
Audible crying duration (s)	3 (0–33)	39 (5–91)	0.0023
Audible crying time ratio (%)	8.3 (0.0–39.8)	44.2 (11.0–73.4)	0.0017
SpO_2_			
Before	100 (99–100)	100 (99–100)	0.38
1 min	99 (95–100)	97.5 (91–100)	0.078
HR			
Prior to heel puncture	128 (121–178)	130 (124–141.25)	0.39
1 min post heel puncture	161 (144–178)	168 (152.75–185.25)	0.053
NIPS score			
Prior to heel puncture	0 (0–0)	0 (0–0)	0.97
At heel puncture	1 (0–5)	5 (3–6)	0.0060
1 min post heel puncture	1.5 (0–6)	5 (0.5–7)	0.098

Data are shown as median (interquartile range) except for re‐puncture.

Abbreviations: HR, heart rate; NIPS, neonatal infant pain scale; SpO_2_, percutaneous arterial oxygen saturation.

The duration of audible crying was significantly shorter in the automatic lancet group (median 3 s, IQR 0–33 s) than in the needle group (median 39 s, IQR 5–91.5 s) (*P *= 0.0023) (Table 2). The audible crying time ratio – the ratio of audible crying time to total treatment time – was also significantly shorter in the automatic lancet group compared to the needle puncture group (median 8.3%, IQR 0.0–39.8% vs median 44.2%, IQR 11.0–73.4%, *P *= 0.0017).

The NIPS scores in the two groups are shown in Table 2. The NIPS score before heel puncture was similar in the automatic lancet group and needle group (median 0, IQR 0–0 vs median 0, IQR 0–0, *P *= 0.97). The NIPS score at the time of puncture was significantly lower in the automatic lancet group than in the needle group (median 1, IQR 0–5 vs median 5, IQR 3–6, *P *= 0.0060) but there was no significant difference at 1 min after puncture (median 1.5, IQR 0–6 vs median 5, IQR 0.5–7, *P *= 0.098) (Table 2).

Heart rate before heel puncture was similar in the automatic lancet and needle puncture groups (median 128 bpm, IQR 109–138 bpm vs median 130 bpm, IQR 124–141.25 bpm, *P *= 0.39). Heart rate at 1 min after the puncture increased from before the puncture in both groups, but the difference did not reach significance (median 161 bpm, IQR 144–178 bpm vs median 168 bpm, IQR 152.75–185.25 bpm, *P *= 0.053). Oxygen saturation (SpO_2_) before heel puncture was similar in the automatic lancet and needle groups (median 100%, IQR 99–100% vs median 100%, IQR 99–100%, *P *= 0.38). Oxygen saturation (SpO_2_) at 1 min after the puncture decreased from before the puncture in both groups, but the difference did not reach significance (median 99%, IQR 95–100% vs median 97.5%, IQR 91–100%, *P *= 0.078). No significant difference was found between the two groups in the number of neonates with heel bruising, inflammation or scar (Table 3).

**Table 3:** Neonatal heel recovery post heel puncture

	Automatic lancet (*n* = 53)	Needle (*n* = 52)	*P* value
Bruising	*n* = 3 (5.7%)	*n* = 6 (12%)	0.47
Inflammation	*n* = 2 (3.8%)	*n* = 0 (0.0%)	0.48
Scar	*n* = 44 (83%)	*n* = 46 (88%)	0.97

## Discussion

This is the first paper comparing the pain response between needle puncture and automatic lancet. Our result uncovered a problem in heel blood sampling by needle puncture.

In this prospective, randomized controlled clinical trial, we compared pain intensity between an automatic lancet and a needle puncture during heel‐prick blood sampling of neonates. Assessment of neonatal pain intensity was based on the audible crying duration, the audible crying time ratio, and the NIPS score. The audible crying duration and the audible crying time ratio were found to be shorter, and the NIPS score lower in the automatic lancet group compared with the needle puncture group.

The results of this study show that use of an automatic lancet for neonatal heel‐prick sampling reduces neonatal pain in comparison to a 23 G needle puncture. In an attempt to assess the pain more accurately, we assessed changes in SpO_2_ and HR. No significant difference was found in either the increase in HR or the decrease in SpO_2_ levels between the two groups. This is consistent with previous reports.^14^, ^18^ These studies measured HR with a pulse oximeter probe, while our study used an electrocardiogram in order to increase the accuracy of the HR measurement. However, there was still no significant difference between the two groups. Although there was no significant difference in change of HR and SpO_2_, an automatic lancet trended to be preferable compared to needle puncture. We suspect that the small number of cases is the reason why a significant difference was not found.

The reduced pain found with automatic lancet use could potentially be explained by its controlled depth of penetration and more superficial plane. Our study found no significant difference between the automatic lancet group and the needle puncture group in the blood collection time, the number of calf squeezes, and the number of re‐punctures. Other studies, however, have reported fewer re‐punctures, calf squeezes and shorter blood collection time with automatic lancets compared to manual lancets in heel blood collection.^10^, ^13^ This study made use of the BD Quick heel lancet, which is the only automatic lancet currently available in Japan. The Tenderfoot lancet has been reported to be superior to other automatic lancets in pain severity, re‐puncture, calf‐squeeze frequency, and blood‐collection time.^19^ It is possible that the difference between the BD Quick heel lancet and the Tenderfoot lancet influenced these results of our study.

The use of an automatic incision device for collecting heel puncture samples has previously been reported to cause less tissue trauma than the use of a conventional manual lancet.^10^, ^12^ Comparison of needle sampling and automatic lancet sampling in this study demonstrated no significant difference in the occurrence of bruising, inflammation, or scars 24 h after heel puncturing. This may be due to the lack of difference between the blood sampling time and the number of squeezes in the needle puncture and automatic lancet groups.

No studies comparing needle and automatic lancet puncture in heel blood sampling have yet been published, and there is a lack of evidence that automatic lancets help to reduce neonatal pain compared to needle puncture. Our study has shown no significant difference in blood collection time, number of calf squeezes, or re‐puncture frequency between the two groups. There is also no difference in ease of blood collection between the two techniques. This study does, however, show reduced pain in the automatic lancet group compared to the needle puncture group.

Automatic lancets are recommended in many developed countries' guidelines^20^, ^21^ and it seems unlikely that a needle would be used to collect heel blood samples in such countries. However, in developing countries, where cost is an issue, needle puncture heel prick tests are more cost effective. The approximate cost of 23‐gauge needle is list price 8.7 yen (0.08 dollar) compared with 150 yen (1.39 dollar) for the automatic lancet. The large price difference between the two devices is a major barrier to the adoption of automatic lancets.

The alleviation of pain at the time of blood collection in neonates is currently an important issue. We believe that this cost difference is justified to improve the prognosis of newborns. Although automatic lancets are recommended in the Japanese guidelines, heel‐blood sampling with a needle is still widely conducted in many NICUs. The authors hope that this article will help to increase the use of automatic lancets at such NICUs to decrease neonatal pain.

A possible limitation of this study is the large number of cases where parental consent for participation in the study was not obtained. Consent was obtained by submitting a research agreement to the parents the day before the newborn screening test, and several parents either forgot or otherwise failed to submit the research agreement. Bias may have occurred due to the number of cases in which consent was not obtained. Another possible limitation is variability in the examiner's technique with either the automatic lancet or the needle. It is possible that the physician carrying out the procedure was more confident in the use of one device. This study also excluded neonates with a birthweight of less than 1,500 g and a gestation of less than 30 weeks. We need to conduct further studies on pain reduction in these premature neonates.

## Conclusion

Heel blood sampling in infants using an automatic lancet was found to be less painful than the needle puncture technique, but there was no difference in blood sampling time, frequency of re‐puncture, or wound healing after puncture.

## Disclosure

The authors declare no conflict of interest.

## Author contributions

Tatenobu Goto, Katsumi Ushijima, Kyoko Ban, and Yasumasa Yamada designed the study. Tatenobu Goto, Takeshi Inoue, Chinami Kamiya, Hiroyuki Kawabe, Machiko Higuchi, Megumi Suyama, Tomoki Goto, Wakato Koide, and Kanemasa Maki collected and analyzed the data. Katsumi Ushijima, Kyoko Ban, and Yasumasa Yamada gave technical support and conceptual advice. Tatenobu Goto performed the statistical analysis and drafted and the manuscript. Yasumasa Yamada critically reviewed the manuscript and supervised the whole study process. All authors read and approved the final manuscript.
